# MG53’s non-physiologic interaction with insulin receptor: lack of effect on insulin-stimulated Akt phosphorylation in muscle, heart and liver tissues

**DOI:** 10.3389/fendo.2024.1425426

**Published:** 2024-09-17

**Authors:** Kyung Eun Lee, Miyuki Nishi, Jongsoo Kim, Takashi Murayama, Zachary Dawson, Xiaoliang Wang, Xinyu Zhou, Tao Tan, Chuanxi Cai, Hiroshi Takeshima, Ki Ho Park

**Affiliations:** ^1^ Division of Surgical Sciences, Department of Surgery, University of Virginia, Charlottesville, VA, United States; ^2^ Department of Biological Chemistry, Graduate School of Pharmaceutical Sciences, Kyoto University, Kyoto, Japan; ^3^ Department of Cellular and Molecular Pharmacology, Graduate School of Medicine, Juntendo University, Tokyo, Japan

**Keywords:** Akt phosphorylation, C2C12 cells, type 2 diabetes mellitus, HepG2 cells, MG53/TRIM72, mice, myokines, insulin receptor

## Abstract

**Rationale:**

MG53’s known function in facilitating tissue repair and anti-inflammation has broad applications to regenerative medicine. There is controversy regarding MG53’s role in the development of type 2 diabetes mellitus.

**Objective:**

This study aims to address this controversy – whether MG53’s myokine function contributes to inhibition of insulin signaling in muscle, heart, and liver tissues.

**Study design:**

We determined the binding affinity of the recombinant human MG53 (rhMG53) to the insulin receptor extracellular domain (IR-ECD) and found low affinity of interaction with K_d_ (>480 nM). Using cultured C2C12 myotubes and HepG2 cells, we found no effect of rhMG53 on insulin-stimulated Akt phosphorylation (p-Akt). We performed *in vivo* assay with C57BL/6J mice subjected to insulin stimulation (1 U/kg, intraperitoneal injection) and observed no effect of rhMG53 on insulin-stimulated p-Akt in muscle, heart and liver tissues.

**Conclusion:**

Overall, our data suggest that rhMG53 can bind to the IR-ECD, however has a low likelihood of a physiologic role, as the K_d_ for binding is ~10,000 higher than the physiologic level of MG53 present in the serum of rodents and humans (~10 pM). Our findings question the notion proposed by Xiao and colleagues – whether targeting circulating MG53 opens a new therapeutic avenue for type 2 diabetes mellitus and its complications.

## 1 Introduction

Since its discovery as a cell membrane repair protein in 2009 (1), MG53/TRIM72 has been the subject of significant research, shedding light on its mechanistic actions in regenerative medicine and its role in regulating metabolic syndromes (2, 3). Beyond its role in tissue repair, MG53 also exhibits anti-inflammatory properties, offering potential applications in aging biology, organ failure, viral infection, and wound healing (4–6). However, the debate surrounding MG53’s involvement in the development of type 2 diabetes mellitus persists (3, 7–12).

Xiao and colleagues proposed that MG53-mediated downregulation of IRS-1 could serve as a causative factor for development of type II diabetes (10, 12). However, other studies demonstrated that even total ablation of IRS-1 is not sufficient to induce type II diabetes in mice (13, 14). Moreover, many other investigators have presented data that challenge their conclusion (15–18). We previously generated db/db mice with knockout or overexpression of MG53 and found no changes in insulin signaling or glucose metabolism, suggesting that “MG53 does not manifest the development of diabetes in db/db mice” (11). Recently, Philouze et al. (9) conducted an extensive series of biochemical and physiological assays and also concluded that “MG53 is not a critical regulator of insulin signaling pathway in skeletal muscle”. In our recent publication in JCI Insight (19), we demonstrated that MG53 suppresses NF-κB activation to mitigate age-related heart failure in mice, and repetitive administration of recombinant human MG53 protein (rhMG53) did not produce adverse effects on major vital organs.

Wu et al. (12) proposed that MG53 has dual actions as a myokine and an E3 ligase to inhibit insulin signaling in muscle, heart, and liver tissues. Here we present new data that found no evidence of a physiologic interaction of MG53 with insulin receptor, and negative result for MG53 in controlling insulin-stimulated Akt phosphorylation in muscle, heart and liver tissues. These findings further challenge the notion proposed by Wu et al. that intervention to reduce circulating MG53 can be beneficial to diabetes and its complications.

## 2 Research design and methods

Biolayer Interferometry (BLI) Assays were utilized to analyze protein binding affinity. The binding affinity of rhMG53 with human insulin receptor extracellular domain (IR-ECD)-His (INR-H52Ha, Acro Biosystems) was determined using Anti-Penta-His (HIS1K) biosensors in an Octet Red 96 instrument (ForteBio Inc., Menlo Park, CA). IR-ECD-His was immobilized onto the surface of HIS1K biosensors. Increasing concentrations of rhMG53 were allowed to interact with the immobilized IR-ECD at room temperature in running buffer (10 mM Hepes, 150 mM NaCl, 0.005% Tween-20 pH 7.4). The final volume of all solutions was 200 µL. Assays were performed in black solid 96-well flat bottom plates on a shaker set at 1,000 rpm/min. The association and dissociation of IR-ECD was measured for 250 sec intervals. All data were analyzed using the Octet Data Analysis 9.0 software (ForteBio).

Cell culture experiments were conducted using C2C12 myotubes and HepG2 cells to investigate the effects of recombinant human MG53 (rhMG53) on insulin-stimulated phosphorylation of Akt (p-Akt). Cell lines were maintained at 37°C, 95% air, and 5% CO2 in a humidified incubator. C2C12 cells (ATCC, CRL-1772) were cultured in Dulbecco’s Modified Eagle’s Medium (DMEM) with 10% fetal bovine serum (FBS, GibcoTM, 16140071) and 1% penicillin-streptomycin (GibcoTM, 15140122). For differentiation, C2C12 cells were cultured in the DMEM with 2.5% horse serum (GibcoTM, 26050088) for 5 days. HepG2 cells (ATCC, HB-8065) were cultured in RPMI1640 medium with 10% FBS and 1% penicillin-streptomycin. HepG2 and differentiated C2C12 cells were pre-treated with 10, 100, or 500 nM of rhMG53 (or 500 nM of BSA as control) for 60 minutes, and then stimulated with 10 nM of insulin (GibcoTM, 12585014) for 10 minutes. Cells were washed using ice-cold PBS and detached mechanically using a scraper, and then collected.

*In vivo* studies were performed on C57BL/6J male mice to assess the impact of intravenous administration of rhMG53 on insulin-stimulated p-Akt levels in muscle, heart, and liver tissues. Animal handling and experimental procedures were performed according to protocols approved by the Institutional Animal Care and Use Committee (IACUC) of University of Virginia and were compliant with guidelines of the American Association for the Accreditation of Laboratory Animal Care. All experimental mice purchased from The Jackson Laboratory were 8-10 weeks of age (C57BL/6J, The Jackson Lab stock No: 000664). For *in vivo* treatment, after fasting overnight (13-15 hours), mice were administered rhMG53 and BSA intravenously at doses of 1 mg/kg and 6 mg/kg, respectively. 10 minutes later, they were subjected to stimulation with 1 U/kg of insulin via intraperitoneal injection. The heart, liver, and skeletal muscles were collected 10 minutes after insulin stimulation, and snap-frozen in liquid nitrogen, and stored in -80°C for later processing and analysis.

Whole lysates were isolated from cells (C2C12 and HepG2) and mouse tissues (heart, liver, and skeletal muscles) with RIPA lysis buffer containing protease inhibitor (Sigma, P8340) and phosphatase inhibitors (Sigma, P0044). The denatured proteins (20 µg/well) were separated by 4-12% protein gel (Thermo Scientific) and transferred onto PVDF membranes (MiliporeSigma, IPVH00010). Membranes were incubated in 5% (w/v) nonfat dry milk for 1 hour at room temperature, further probed with primary antibody and incubated at 4°C with gentle shaking overnight. Then, they were washed with Tris-buffered saline with 0.1% Tween 20 detergent (TBST) and probed with a HRP (horseradish peroxidase)-conjugated secondary antibody. Immunodetection was performed by iBrightTM CL 1500 Imaging System (Invitrogen) using SuperSignal West Pico PLUS Chemiluminescent Substrate (Thermo Scientific, 34580). Primary antibodies used were as follows: Akt (Cell Signaling Technology, 9272S, 1:1000 dilute), phospho-Akt (Ser 473) (Cell Signaling Technology, 4060S, 1:2000 dilute), IRS-1 (Cell Signaling Technology, 2382S, 1:1000 dilute), phospho-IRS-1 (Ser 636/639) (Cell Signaling Technology, 2388S, 1:1000 dilute), anti-MG53 (custom-made, 914, 1:2000), GAPDH (Cell Signaling Technology, 2118S, 1:5000 dilute) was diluted in 2.5% (w/v) nonfat dry milk.

ANOVA test (single factor) as statistical analysis was used to determine statistical difference between groups. All data were analyzed using Excel and GraphPad Prism 10 software.

## 3 Results and discussion

Wu et al. (12) reported elevated serum levels of MG53 (~2 fold) in diabetic mouse models and human diabetic patients, concluding that MG53 has dual actions as a myokine and an E3 ligase to inhibit insulin signaling in muscle, heart and liver tissues. Using BiACore assay, they showed recombinant human MG53 (rhMG53) protein binds to the extracellular domain of insulin receptor (IR-ECD) to block insulin signaling, with an estimated K_d_ of 8 nM [Figure 6C Wu et al (12)]. Compared to the rapid k_off_/k_on_ of insulin binding, the kinetics for rhMG53 binding to IR-ECD is atypical; rhMG53 has slow reaction and fails to reach equilibration or saturation [Figure 6B Wu et al. (12)].

**Figure 1:**
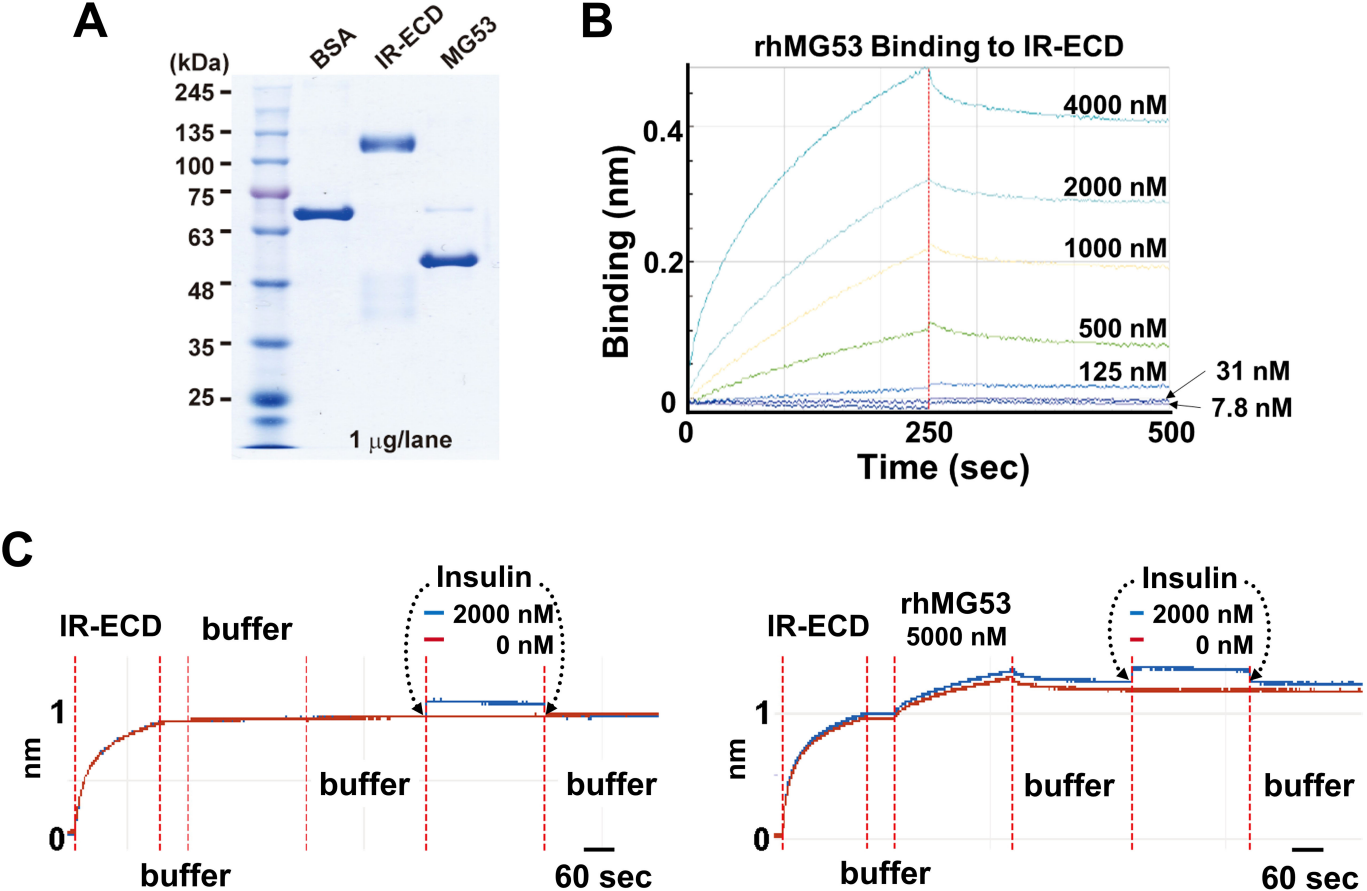
Biolayer interferometry (BLI) assays. **(A)** SDS-polyacrylamide gel electrophoresis (SDS-PAGE) of bovine serum albumin (BSA, Sigma A1470), IR-ECD (Acro Biosystem), and rhMG53 (see ref (2) for preparation). **(B)** In the protein-protein interaction assay using Octet RED96 instrument (ForteBio Inc., Menlo Park, CA), Octet sensorgrams of rhMG53 binding to IR-ECD revealed a K_d_ > 480 nM (n=3). **(C)** BLI assay of insulin binding to IR-ECD without rhMG53 (left panel) or with 5,000 nM rhMG53 preloaded onto the biosensor chip (right panel). Insulin binding to IR-ECD was not affected by rhMG53.

We have followed the exact protocol as described and found that rhMG53 can interact with IR-ECD but at a much higher K_d_ (>480 nM) (**Figures 1A, B**). Moreover, we confirmed pre-loading of the Octet sensorgrams with rhMG53 does not impact insulin’s affinity with IR-ECD (**Figure 1C**). Our results indicate that rhMG53 does not directly interfere with insulin binding to IR-ECD, and rhMG53 has a much lower competitive/allosteric threshold than previously described.

**Figure 2:**
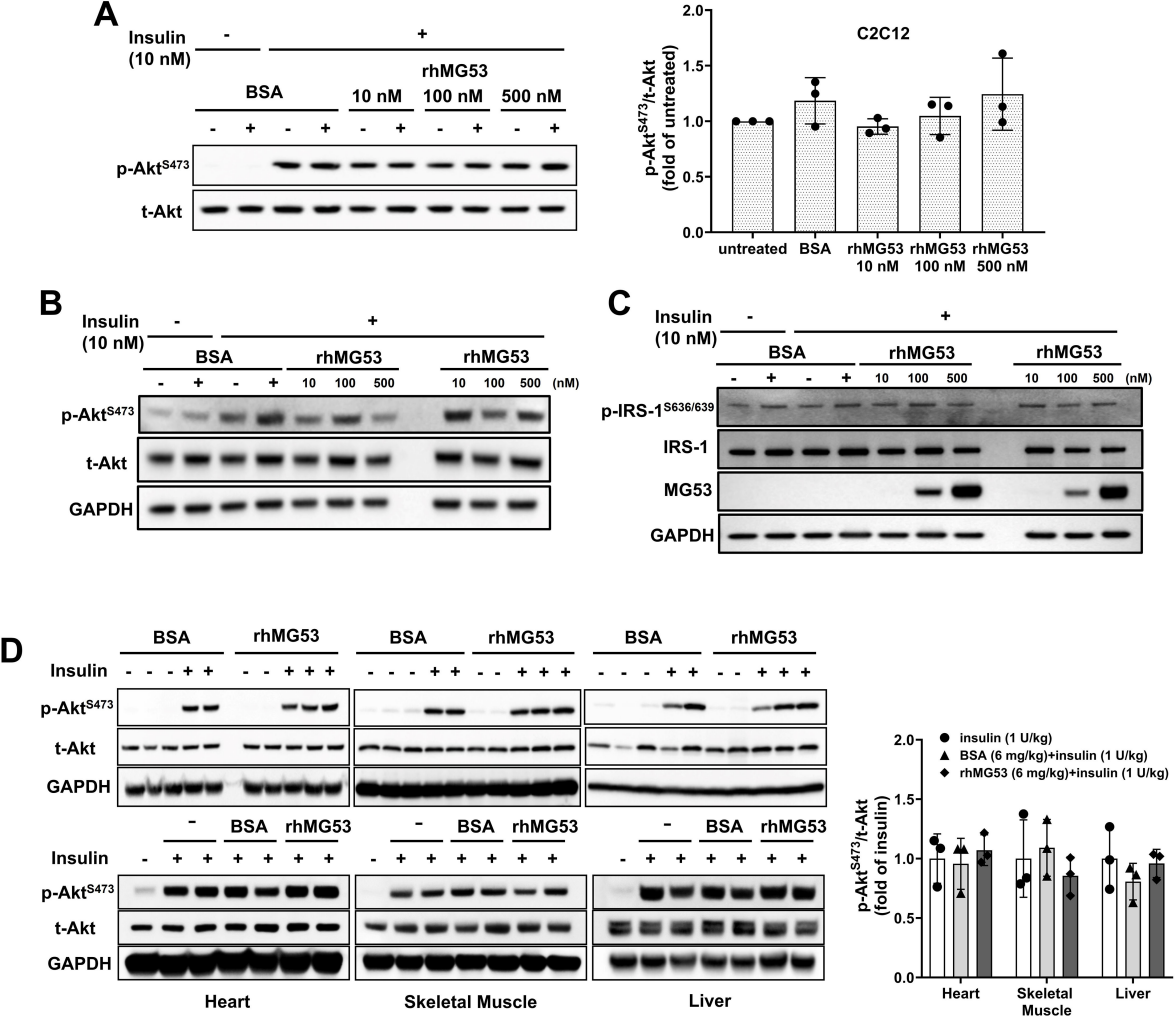
Role of MG53 in insulin signaling pathways in C2C12 myotubes, HepG2 cells, and mouse tissues. **(A)** C2C12 myotubes (5 days post differentiation) (n=3) and **(B)** HepG2 cells were treated with varying concentrations of rhMG53 for 1 hour (or BSA as control), followed by 10 nM insulin for 10 min. Western blot were conducted with antibody against p-Akt [Cat #4060, Cell Signaling Technology (CST)] and total Akt (t-Akt) (Cat #9272, CST) (n=2). **(C)** HepG2 cells were treated with varying concentrations of rhMG53 for 1 hour (or BSA as control), followed by 10 nM insulin for 10 min. Western blot were conducted with antibody against total IRS-1 (Cat #2382S, CST), p-IRS-1 (Cat #2388S, CST), anti-MG53 (custom-made, 914), GAPDH (Cat #2118S, CST) (n=2). **(D)** 1 mg/kg rhMG53 (upper panel) and 6 mg/kg rhMG53 (lower panel) treatment did not alter the level of p-Akt in skeletal muscle, heart and liver derived from mice subjected to 1 U/kg insulin treatment. Sample sizes were as follows: 1 mg/kg BSA + 1 U/kg insulin (n=2), 1 mg/kg rhMG53 + 1 U/kg insulin (n=3); 1 U/kg insulin (n=3), 6 mg/kg BSA + 1 U/kg insulin (n=3), 6 mg/kg rhMG53 + 1 U/kg insulin (n=3).

Wu et al. (12) further suggested that “… extracellular MG53 acts as an allosteric, rather than a competitive, blocker of the IR”, based on their findings in liver cell culture where incubation with rhMG53 (not BSA) inhibited insulin-stimulated phosphorylation of Akt (p-Akt) [Figure 7A Wu et al. (12)]; and intravenous administration of rhMG53 blocked insulin-stimulated p-Akt in mouse tissues [Figure 5A Wu et al. (12)]. We were puzzled by these results, as a recent report by Philouze et al. (9) showed negative findings with rhMG53 on insulin-stimulated p-Akt [Figures 3, 4 Philouze et al. (9)]. Thus, we conducted a study to determine whether rhMG53 alters insulin-stimulated p-Akt in a dose-dependent manner. We treated C2C12 myotubes and HepG2 cells with 10 nM of insulin, then quantified the impact of rhMG53 in a wide range of concentrations (10, 100 and 500 nM). As shown in **Figures 2A, B**, we saw no effect of rhMG53 on insulin-stimulated p-Akt in all concentrations tested.

While our previous study (17) and study from other investigators (10) have found physical interaction between MG53 and IRS-1, and MG53 can facilitate ubiquitination of IRS-1, there is discrepancy on the *in vivo* role of MG53-mediated degradation of IRS-1 in muscle and heart tissues (8, 11). Our tests showed rhMG53 does not appear to impact the protein level for IRS-1 or insulin-stimulated IRS-1 phosphorylation (**Figure 2C**).

These findings prompted us to conduct *in vivo* assays to determine whether intravenous administration of rhMG53 has any impact on insulin-stimulated p-Akt in muscle, heart and liver tissues. Following the protocol of Wu et al. (12), C57BL6J mice (male, 8-10 weeks old, The Jackson Laboratory) were treated with 1 U/kg of insulin (intraperitoneal injection); 10 min prior to insulin injection, 1 mg/kg or 6 mg/kg of rhMG53 was administered into the tail vein of the mice. Administration of 1 mg/kg rhMG53 would result in a peak level of MG53 in the blood (~10 µg/ml) which should be 1000-fold and 500-fold higher than the resting level of MG53 observed in healthy and diabetic animals. We would also expect this to further saturate the IR-ECD if any allosteric interaction was occurring. However, we were surprised to find that rhMG53 has no impact on p-Akt level in skeletal muscle, heart or liver tissues (**Figure 2D**), which contrasts the data presented by Wu et al. (12), who notably used 6 mg/kg of rhMG53 in their study.

Overall, our data suggest that rhMG53 can bind to IR-ECD, however has low likelihood of a physiologic role, as the K_d_ for binding is ~10,000 higher than the physiologic level of MG53 present in the serum of rodents and humans (~10 pM). Consistent with the data shown by Wu et al. (12), rhMG53 clearly does not interfere with insulin binding at the IR. Based on our current findings and those of Philouze et al. (9), it is unlikely that MG53 has any allosteric impact on insulin-signaling in muscle, heart and liver tissues. All together, these findings question the notion proposed by Wu et al. (12) – whether targeting circulating MG53 opens a new therapeutic avenue for type 2 diabetes mellitus and its complications.

## Data Availability

The original contributions presented in the study are included in the article/supplementary material. Further inquiries can be directed to the corresponding authors.
